# Electrolyte Design for Lithium Metal Anode‐Based Batteries Toward Extreme Temperature Application

**DOI:** 10.1002/advs.202101051

**Published:** 2021-07-17

**Authors:** Dan Luo, Matthew Li, Yun Zheng, Qianyi Ma, Rui Gao, Zhen Zhang, Haozhen Dou, Guobin Wen, Lingling Shui, Aiping Yu, Xin Wang, Zhongwei Chen

**Affiliations:** ^1^ School of Information and Optoelectronic Science and Engineering & International Academy of Optoelectronics at Zhaoqing South China Normal University Guangzhou 510006 China; ^2^ Department of Chemical Engineering Waterloo Institute of Nanotechnology University of Waterloo Waterloo N2L 3G1 Canada

**Keywords:** electrolyte, extreme temperature, lithium metal batteries, solid electrolyte interface

## Abstract

Lithium anode‐based batteries (LBs) are highly demanded in society owing to the high theoretical capacity and low reduction potential of metallic lithium. They are expected to see increasing deployment in performance critical areas including electric vehicles, grid storage, space, and sea vehicle operations. Unfortunately, competitive performance cannot be achieved when LBs operating under extreme temperature conditions where the lithium‐ion chemistry fail to perform optimally. In this review, a brief overview of the challenges in developing LBs for low temperature (<0 °C) and high temperature (>60 °C) operation are provided followed by electrolyte design strategies involving Li salt modification, solvation structure optimization, additive introduction, and solid‐state electrolyte utilization for LBs are introduced. Specifically, the prospects of using lithium metal batteries (LMBs), lithium sulfur (Li‐S) batteries, and lithium oxygen (Li‐O_2_) batteries for performance under low and high temperature applications are evaluated. These three chemistries are presented as prototypical examples of how the conventional low temperature charge transfer resistances and high temperature side reactions can be overcome. This review also points out the research direction of extreme temperature electrolyte design toward practical applications.

## Graphical abstract

This review introduces current progress of electrolyte design in lithium metal batteries to realize improved performance under extremely low and high temperature applications. By reviewing the scientific issues related to the current electrolyte design strategy, including Li salt modification, solvent component optimization, electrolyte additive introduction and solid‐state electrolyte utilization, this review points out the research direction toward practical applications.

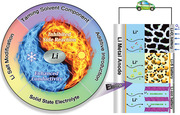

## 1 Introduction

With the ever‐increasing energy storage system demands, lithium‐ion technologies are likely unable to meet further increases in requirements due to its limited theoretical capacity and practical energy density at the cell level.^[^ ^1^ ^]^ Lithium metal anode based batteries (LBs) have attracted much attention in the scientific community owing to the high theoretical capacity (3860 mAh g^−1^), low reduction potential (−3.04 V vs. SHE, standard hydrogen electrode) and ideally host‐less nature of lithium (Li).^[^ ^2^ ^]^ Using lithium metal as the anode can be considered as an ultimate goal for improving the energy density limit of rechargeable battery.^[^ ^3^ ^]^ The emerging LBs comprise of three types of batteries which all employed lithium metal anode (LMA) with different cathodes: lithium metal batteries (LMBs) with an intercalation‐type lithiated metal oxide as cathode material, lithium‐sulfur (Li‐S) batteries with S composite as cathode material and lithium‐oxygen (Li‐O_2_) batteries with O_2_ as cathode material.^[^ ^4^ ^]^ These battery systems have aroused significant interest as promising sustainable energy‐storage systems. However, the biggest challenges for LBs, especially for the aforementioned LBs with potentially high energy density, are the significant performance degradation and/or decreased safety under extreme temperature range (below 0 °C and above 60 °C), which are common operating conditions in battery applications such as portable electronics, stationary energy storage or electric vehicles (EVs) over seasonal changes in weather or overuse. To overcome these challenges, the battery research communities have placed numerous efforts toward investigating the fundamentals of these systems and developing various strategies relevant with critical components such as electrolyte, electrode and their interface to improve the performance and stability of LBs at such extreme temperature range.^[^ ^5^ ^]^ Comparatively, the electrolyte relevant which is usually based on organic liquid, is more affected by extreme temperature operation and further impacts on battery performance.

Specifically, the sharp decline in cell output at subzero temperatures is the combined consequence of the decreased capacity utilization and depressed cell potential raised by the retarded ion transport in bulk electrolyte solutions, the sluggish solvation/desolvation process of Li^+^ and exponentially increased interfacial charge transfer resistance on cathode and anode, which result in a significantly decreased energy output. On the other hand, when operating the LBs under high service‐temperature, the thick solid electrolyte interface (SEI) formation induced by severe electrolyte reduction leads to huge polarization and poor cycling performance of LBs.^[^ ^6^ ^]^ Meanwhile, the low flash point of ether/ester solvents is prone to be ignited under elevated temperature, promoting dangerous battery fire or even explosion. Therefore, searching for optimized electrolytes with high compatibility and electrochemical stability, satisfied ionic conductivity, desired SEI formation capability, wide service‐temperature range, high safety and low cost are indispensable for the development of high‐energy‐density batteries.

**Figure 1:**
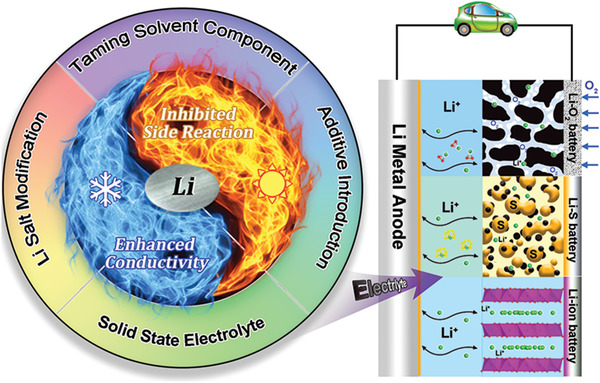
Summary of electrolyte design in Li metal anode‐based batteries for extreme temperature operation.

To facilitate research and development in overcoming these challenges, we have organized this review to bring forward extreme temperature electrolyte design strategies of LBs, which is rarely summarized in current publications. Research into widening the working temperature of electrolyte has become a crucial topic identified as a pathway to solve the bottleneck problems of LBs for the practical applications. This review paper will introduce the fundamentals of electrolyte design principle in LBs, especially for LMB with intercalation‐type lithium‐containing transitional metal oxide cathode material and conversion‐type lithium‐chalcogenide batteries with S or O_2_ as cathode material, as shown in **Figure** **1**. It aims to summarize the pivotal scientific issues related to the current electrolyte design strategy for low and high temperature operation scenario, which involves Li salt modification, solvent component optimization, electrolyte additive introduction and solid‐state electrolyte utilization. Then it will provide meaningful perspectives for the future development of extreme temperature electrolytes and point out the research direction of electrolyte design toward practical applications.

## 2 Fundamental and Challenge in Electrolyte Design

In a battery, the chemical nature of cathode and anode decides the energy output, while the electrolyte has substantial impacts on ion/mass transportation for charge/discharge process. This in turn determines how fast the energy could be released by controlling the rate of mass flow within the battery. Additionally, the voltage window of the electrolyte mostly limits the selection of anode and cathode. The following requirements should be considered when choosing an electrolyte for LBs. First, the electrolyte should exhibit good ionic conductivity and electronic insulating property to ensure fast ion transfer without self‐discharge. It should also have a good electrochemical stability without electrolyte degradation within the range of the working potentials. The electrolyte should also be inert to other cell components such as cell separators, electrode substrates, and cell packaging materials.

### 2.1 Ion Transfer Kinetics of Electrolyte

Understanding the Li‐ion transportation kinetics has substantial impacts on the design of electrolyte to enhance the redox reaction kinetics, especially under extreme temperature conditions. The theoretical ion conductivity behavior (*κ*) in typical electrolyte can be calculated from Stokes law, as shown in Equation 1:

(1) $$\kappa =\sum_{i}\frac{{\left(Z_{i}\right)}^{2}FC_{i}}{6\pi r_{i}}$$

where *Z_i_* is charge number in the charge transfer process, *C_i_* is molar concentration, *F* is the Faradaic constant, *η* is the viscosity and *r_i_* is the radius of solvation ions. Clearly, the ionic conductivity is highly dependent on the salt concentration and electrolyte viscosity. To achieve a high Li salt concentration, the solvents should have a high dielectric permittivity (*ε*) to sufficiently dissociate and decouple the Li ions from the anions and offer more Li^+^ ions for charge transfer. High dielectric constant solvent is able to preferentially interact with Li^+^ ions, which facilitates the dissolution and dissociation of lithium polysulfides (LiPSs) discharge intermediates in liquid electrolyte and reduces electrolyte viscosity, ensuring a fast charge transfer process. In Li‐S batteries, the high content of high dielectric constant solvent in the Li^+^ solvation structure also suppresses the association of polysulfide anion and Li^+^ in the electrolyte, avoiding the precipitation of LiPSs and further expose the inner part of S particle during discharge process. However, increasing the dielectric constant of solvent also tend to increase the electrolyte viscosity, which is detrimental for ion transportation. Since a higher dielectric constant means a stronger affinity between the solvent molecules and the Li^+^, the solvation process is greatly promoted. However, the de‐solvation process on the electrode surface is suppressed, limiting the Li‐ion intercalation kinetics on the cathode. On the anode side, the higher dielectric constant solvent provides stronger interaction with Li^+^, which results in surface depletion of Li^+^ during deposition and induces severe Li dendrite growth. Therefore, the practically viable LBs with desirable electrochemical performance can only be realized by making tradeoff between dielectric constant and viscosity of solvent.

### 2.2 Interfacial Stability of Electrolyte

While the potencies of electrode materials are usually quantified by the redox potential in volts against some certain reference potential, the stability of an electrolyte can also be quantified by the range in volts between its oxidative and reductive decomposition limits, which is known as the “electrochemical window.” Obviously, the redox potential of both electrode materials must fall within this electrochemical window to enable a rechargeable battery operation. According to the frontier molecular orbital theory, a molecule with a comparatively lower lowest unoccupied molecular orbital (LUMO) is comparatively easier to be reduced, while a molecule with a higher highest occupied molecular orbital (HOMO) is will be oxidized. Suppose that the electrochemical potentials of anode and cathode are *µ*_A_ and *μ*_C_, respectively.^[^ ^7^ ^]^ *E*_LUMO_ and *E*_HOMO_ refer to the voltages corresponding to LUMO and HOMO. If *µ*_A_ > *E*_LUMO_, electrons on the anode are inclined to transfer to the unoccupied orbital of the electrolyte, inducing the intrinsic reduction reactions of electrolyte by forming SEI on the anode. Similarly, in the case of *μ*_C_ < *E*_HOMO_, redox reactions contribute to the generation of cathode electrolyte interphase (CEI) between electrolyte and cathode.

Ideally, the electrolyte could be viewed as the inert component with excellent stability against both cathode and anode surfaces. However, it is often challenged by the strong oxidizing and reducing nature of the cathode and the anode, respectively. Practically, due to the most negative nature of the electrochemical potential of Li, the redox reactions between Li and electrolyte cannot always be avoided. The severity of this challenge is the ever‐increasing pursuit of new battery systems with higher energy densities. Therefore, understanding the electrolyte/electrode interfacial phenomenon from thermodynamic and kinetics aspects is able to illuminate the underlying mechanism for the side reactions and their impact on the charge transportation on the interfaces.

The spontaneous reaction between electrolyte and highly reductive metallic Li induces electrically insulating and ionically conductive SEI formation on the interface by the parasitic reactions. If SEI formation were sustained throughout battery operation, it would render anode unusable due to the continual loss of Li. Fortunately, the SEI film can physically block the electrolyte contact with LMA and conscientiously protect Li metal to avoid its further reduction. Once an initial SEI layer has formed, the inability of electrolyte molecules to travel through the SEI to the active material surface, where they could react with Li ions and electrons, suppresses further SEI growth. This formed SEI enable the perfect operation of conventional LIBs, which employs graphite as anode, with long cyclic stability.^[^ ^8^ ^]^ However, in LBs, the Li ions strip from LMA during charge process and plate on LMA during discharge process, which results in huge volume fluctuation and non‐uniform surface deposition.^[^ ^9^ ^]^ Ideally, the LMA should have less than 100% excess of Li in most of LMBs, this requirement endows the anode with a huge volume expansion over 100%. The recurring stripping‐plating process of Li anode results in SEI breakage and fresh Li exposure, which continuously consumes Li and electrolyte, leading to SEI accumulation and electrolyte drying. As for Li‐S batteries, the complexity of cathode reaction induces numerous discharge intermediates formation, which is able to dissolve into electrolyte, diffusing from cathode to anode, induces severe side reactions with Li.

The generally accepted description of the SEI structure is the mosaic model, indicating the surface is not homogeneous. The formation of SEI involves several reductive decompositions proceed on the negatively charged anode surface, rendering the mixture of insoluble multiphase products deposits on the anode.^[^ ^10^ ^]^ The SEI layer in thickness direction usually exhibits a dual‐layer structure, in which the layer close to the Li metal surface contains inorganic Li compound including Li_2_O, Li_3_N, LiF, LiOH, and Li_2_CO_3_ while the outer part of the surface films is comprised of organic Li compound, such as ROCO_2_Li, ROLi, and RCOO_2_Li (R is an organic group related to the solvent).^[^ ^11^ ^]^ However, under low temperature condition, the charge transfer of SEI is greatly impeded, leading to sluggish redox reaction kinetics.^[^ ^12^ ^]^ The SEI film has generally been recognized as the most resistive component in the journey of the Li‐ion transportation.^[^ ^13^ ^]^ An increased *R*_ct_ can be observed as the temperature decreased below zero, indicating the sluggish charge transportation on SEI.^[^ ^14^ ^]^ The interfacial and bulk impedances rapidly increased with the decrease of temperature. The *R*_ct_ can increase up to 10 times when the operation temperature decreases 20 °C for both carbonate‐based and ether‐based electrolyte.^[^ ^15^ ^]^ Thenuwara et al. observed a low *R*_ct_ of 70 Ω at 20 °C in DOL/DME‐10%FEC electrolyte, which significantly increased to ≈10^6^ Ω at −20 °C. The slow charge transportation process further induces non‐uniform Li plating and severe dendrite growth toward cathode, affording the cell with short lifespan and safety issues. As reported by Xu et al.^[^ ^16^ ^]^, the Faradic current in Li plating process can turn to be 0 at low temperature, rendering the Li‐ion depletion on the electrode surface and sluggish kinetics. On the other hand, the SEI undergoes fast degradation and decomposition under elevated temperature, leading to fast electrode failure. Typical SEI degradation pathways are partial dissolution at high temperature or cracking formation due to mechanical stresses inherent to electrode operation. The exposed region further reacts with electrolyte and enhances electrode resistivity. Thermal breakdown of the SEI will occur under extreme temperature, which ultimately yields to thermal runaway.^[^ ^6a^ ^]^ Since the thermodynamically favorable SEI formation on the anode is inevitable, designing a stable SEI by regulating the interfacial reaction is critical for Li metal protection. Recently, fluoride component has been employed to construct stable SEI layer for LMA. Zhang et al.^[^ ^17^ ^]^ employed trifluoromethyl functional groups (─CF_3_) in SEI to tune the orbital energies and the HOMO‐LUMO gap, which inhibits the continuously electrolyte decomposition on the interface. Zhang et al.^[^ ^18^ ^]^ also introduced FEC component in electrolyte to construct a compact LiF‐rich SEI for stable Li stripping/plating process on the anode. However, the underlying scientific mechanism for the formation of dual layer SEI structure is still not fully understood.

### 2.3 Key Component Used in Electrolyte for LBs

#### 2.3.1 Solute

The ideal Li salts should be able to completely dissolve and dissociate in electrolyte and the solvated Li^+^ should have high mobility for ion transportation. Meanwhile, the anion should be stable against oxidative decomposition at the cathode and reduction decomposition at the anode. Most Li salts fail to meet the minimum solubility requirement in low dielectric media due to their small ionic radius of Li^+^. To enhance its solubility, a complex Lewis‐base anion with large radius should be employed. Conventional and widely commercialized carbonate‐based electrolytes for LIBs typically contain lithium hexafluorophosphate (LiPF_6_) as Li salt. Owing to its well‐balanced properties, LiPF_6_ wins out over other Li salts such as lithium perchlorate (LiClO_4_), lithium hexafluoroarsenate (LiAsF_6_), and lithium tetrafluoroborate (LiBF_4_). However, the successful implement of carbonate electrolyte in LIBs is unable to directly copy to LBs. The LMA operated in carbonate electrolyte exhibits low Coulombic efficiency (CE) and severe Li dendrite growth over cycling. In addition, the severe side reaction of conversion‐type LBs between chalcogenides cathode and electrolyte results in limited capacity and low electrochemical reversibility of Li‐S batteries and Li‐O_2_ batteries. Besides, LiPF_6_ is still not the ideal Li salts owing to its poor thermal stability and high moisture sensitivity.^[^ ^19^ ^]^ As a result, the Li salt decomposition generate HF species, which induce the destruction of SEI and CEI on electrodes, the dissolution of transition metals from cathode material, and reductive/oxidative decomposition of solvents. To alleviate these problems, ether solvent and Lithium bis(trifluoro‐methanesulfonyl)imide (LiTFSI) salt were introduced as electrolyte. A more conductive and stable SEI can be formed in this type of electrolyte owing to the Li_2_S and Li_3_N formation in SEI layer induced by LiTFSI decomposition. Recently, lithium bis(fluorosulfonyl)imide (LiFSI),^[^ ^20^ ^]^ lithium (difluoromethanesulfonyl)(trifluoromethanesulfonyl)imide (LiDFTFSI)^[^ ^21^ ^]^ and LiFSI‐LiNO_3_^[^ ^22^ ^]^ were further employed as Li salts in electrolyte, a more stable SEI can formed by modulating the Li^+^ solvation structure. Therefore, anion structure modification strategies formulate complex reduction reactions on Li metal during electrochemical process, which manipulate the structure and component of SEI, rendering enhanced stability and improved kinetics in LBs.

#### 2.3.2 Solvent

The liquid range of a nonaqueous electrolyte system is defined at the upper limit by the temperature at which one of its components begins to vaporize (boiling temperature, *T*_b_) and at the lower limit by the temperature at which one of its components begins to crystallize (melting temperature, *T*_m_). Apparently, this range could serve as the main basis for estimating the operating limits of LBs that employ such an electrolyte system to realize its application in wide‐service temperature. The *T*_b_ and *T*_m_ of frequently used carbonate solvent is presented in **Table** **1**. The commonly used ethylene carbonate (EC) is indispensable in almost all LIB electrolytes since it has high dielectric permittivity and it is capable of forming stable SEI on graphite anode. Other acyclic carbonate or carboxylic esters, such as dimethyl carbonate (DMC), diethyl carbonate (DEC), or ethyl methyl carbonate (EMC), were mixed with EC to reduce the *T*_m_ and facilitate ion transportation in electrolyte. However, this mixed carbonate is unable to from a stable SEI to efficiently protect LMA. Severe dead Li formation and Li dendrite growth can be observed by cycling LMA in carbonate electrolyte.^[^ ^23^ ^]^ For practically viable LBs, the CE should exceed over 99.8% in each cycle to ensure a stable operation over 500 cycles under a negative and positive capacity (N/P) ratio of 2.8. However, a low average CE of ≈92% and poor cycle life of 12 cycles can be obtained in EC/DEC based electrolyte under this scenario, which is still far away from the practical application of LMBs.^[^ ^24^ ^]^ Introducing fluorinated carbonated solvent has been deemed as the appropriate strategy for stable stripping/plating process on LMA. Fluoroethylene carbonate (FEC) was first employed as co‐solvent to stabilize LMA to realize long term cyclic stability. The as‐developed FEC/DMC,^[^ ^25^ ^]^ and FEC/DEC/DME^[^ ^26^ ^]^ based electrolyte exhibit prolonged cycle life and improved CE comparing with conventional EC based carbonate electrolyte. However, the high viscosity of FEC (3.33 cP, 25 °C) endows the electrolyte with limited ion mobility, which exhibits large overpotential during charge‐discharge process. Therefore, mixed fluoride carbonate solvent such as introducing difluoroethylene carbonate (DFEC)^[^ ^27^ ^]^ or *γ*‐butyrolactone (GBL)^[^ ^28^ ^]^ into FEC was developed, which becomes a popular strategy to realize a high CE and excellent cyclic stability for LMBs.

**Table 1:** Physical properties of carbonate solvent

Solvent	Melting temperature *T*_m_ [ °C]	Boiling temperature *T*_b_ [ °C]	Viscosity *η* [cP]	Dielectric constant *ε*	Flash point *T*_f_ [ °C]	Density *ρ* [g cm^−3^]
EC (ethylene carbonate)	36.4	248	1.9	89.78	160	1.32
PC (propylene carbonate)	−48.8	242	2.53	64.9	132	1.2
DEC (diethyl carbonate)	−74.3	126	0.75	2.81	31	0.97
DMC (dimethyl carbonate)	4.6	91	0.59	3.11	0.76	1.06
EMC (ethyl methyl carbonate)	−53	110	0.65	2.96	23	1.01
FEC (fluoro‐ethylene carbonate)	20	210	3.33	78.4	102	1.45
EA (ethyl Acetate)	−84	77	0.45	6.02	−4	0.9
BA (butyl Acetate)	−78	126	0.685	5.1	22	0.882
EB (ethyl butyrate)	−91.5	164	0.639	5.1	25.6	0.829
MP (methyl propionate)	−87.5	79.8	0.431	6.2	−2	0.915
MB (methyl butyrate)	−85.8	102.8	0.541	5.48	12	0.898
EP (ethyl propionate)	−73	99	0.494	5.7	12	0.888
PB (propyl butyrate)	−95.2	143	0.781	4.3	N/A	0.873
BB (putyl butyrate)	−91.5	164	0.876	4.39	49	0.829

In view of the poor cycling efficiency and the potential hazards associated with side reaction of carbonate solvent, ether solvent is widely used for conversion‐type LBs. Comparing with carbonate solvent, the ether solvent possesses much lower viscosity and dielectric constant, ensuring the ether‐based electrolyte with high ion mobility and wettability for facilitated charge/mass transportation. The frequently used solvent in Li‐S and Li‐O_2_ batteries are presented in **Table** **2**. In Li‐S batteries, an improved Li^+^ diffusion coefficient and enhanced cyclic stability can be observed when cycled in ether based electrolyte.^[^ ^29^ ^]^ Interestingly, the S species in electrolyte is preferentially reduced rather than DOL or DME molecule, which contributes to the stable SEI formation and significantly improves the CE of Li‐S batteries.^[^ ^30^ ^]^ Besides, a much reduced electrolyte decomposition on LMA can also be observed. The LUMO energy data obtained by theoretical DFT calculation revealed that ether solvent such as DOL and DME exhibit a much lower LUMO energy than carbonate solvent, indicating the inhibited solvent reduction from the thermodynamic point of view.^[^ ^31^ ^]^ A dendrite‐free deposition morphology and suppressed electrolyte consumption can be observed by using long chain glyme, holding great promises to promote the practical application of Li‐S batteries^[^ ^32^ ^]^ However, the dissolution and dissociation of LiPS in ether solvent results in viscosity increasing of electrolyte during discharge and charge process. This S species can migrate from cathode to anode and significantly reduce the CE of Li‐S batteries, which is known as the notorious shuttle effect. The spontaneously reduction reaction of LiPS will occur on LMA when it diffused to anode, rendering capacity loss and severe LMA corrosion. To further enhance the anode stability, fluoride ether‐based electrolyte was developed and the Li‐S performance was investigated. Cao et al.^[^ ^33^ ^]^ reported the ether electrolyte by using DOL and 1,1,2,2‐tetrafluoroethyl‐2,2,3,3,3‐pentafluoropropyl ether (TPE) as solvent for Li‐S batteries and an admirable CE above 98% and improved capacity retention can be realized. On the other hand, the low flash point of ether solvent pose long‐standing challenges in electrolytes, which limits its liquid temperature range and induces safety concerns. Therefore, developing new type of safe ether solvent which can not only facilitate the Sconversion reaction on the cathode side but also can inhibit electrolyte consumption and Li dendrite growth on the anode side is urgently needed in this area.

**Table 2:** Physical properties of ether and other solvent

Solvent	Melting point *T*_m_ [ °C]	Boiling point *T*_b_ [ °C]	Viscosity *η* [cP]	Dielectric constant *ε*	Flash point *T*_f_ [ °C]	Density *ρ* [g cm^−3^]
DOL (1,3‐dioxolane)	−97.2	75.6	0.6	6.74	1	1.06
DME (1,2‐dimethoxy ethane)	−58	82.5	0.45	5.5	0	0.86
DEGDME (diethyl carbonate)	−64	162	1.88	7.23	57	0.94
TEGDME (ethyl methyl carbonate)	−45	216	2.73	7.9	106	0.99
THF (tetrahydrofuran)	−108.5	66	0.46	7.58	−14	0.88
(2MeTHF) 2‐methyltetrahydrofuran	−75	78	0.46	6.2	−11	0.86
DMSO (dimethyl sulfoxide)	18.5	189	2.0	46.7	88.9	1.1
Sulfolane	27.5	285	0.01	44	165	1.26
DMF (dimethyl‐formamide)	−60.4	153	0.8	37	58	0.94
CCl_4_ (carbon tetrachloride)	−22.9	76.8	0.9	2.2	−0.3	1.58
CS_2_ (carbon disulfide)	−111.9	46.3	0.37	2.6	−43	1.26
ACN (acetonitrile)	−43.8	81.6	0.34	37.5	2	0.78
Acetone	−94.7	56.3	2.7	20.7	−20	0.79
CHCl_3_ (chloroform)	−63.5	61.2	0.54	4.8	N/A	1.48

The solvent selection of Li‐O_2_ battery is much more selective because the carbonate electrolyte is sparsely used as it is susceptible to attack by superoxide anions. This irreversible process results in electrolyte decomposition and might also provide misleading charge potentials. Although a highly reversible reaction can be observed in ether‐based electrolyte, the fast solvent volatilization of low *T*_b_ ether solvent endows the battery with very short calendar life. Also, low *T*_b_ ether solvent usually exhibits a low flash point (*T*_f_), which means that the vaporized solvent molecule in the O_2_‐filled battery is easy to be ignited and induces severe safety issues. On the other hand, utilizing high *T*_b_ ether solvent as electrolyte component is also unsatisfactory since the long chain glyme demonstrates high viscosity with reduced Li‐ion mobility and sluggish reaction kinetics, some of them are even in solid state and unable to transfer Li^+^ under room temperature condition. Trade‐off analysis should be made to select a solvent with appropriate *T*_m_, *T*_b_, *T*_f_ and *η*. Normally, only TEGDME and DMSO based electrolyte are widely reported in Li‐O_2_ batteries. However, these two types of electrolyte hardly induce stable SEI formation and suppress electrolyte volatilization to ensure long cycle life and calendar life for practical application.

## 3 Strategies for Low Temperature Electrolyte Design

At low temperatures, the rapid increasing of viscosity negatively affects ion mobility and electrode wettability. The sluggish mass/charge transportation and volumetric changes raised by electrolyte freezing limit the low‐temperature performance of LBs.^[^ ^34^ ^]^ In order to design electrolytes with high conductivity, the solvents should possess a combination of several critical properties, such as high dielectric constant, low viscosity, adequate coordination behavior, as well as appropriate liquid ranges and salt solubility in the medium. However, the high dielectric constant of the solvents inevitably enhances the dipole‐dipole force among these highly polar molecules, increasing the freezing temperature of the solvents and thus reducing the low‐temperature performance of the electrolytes. With respect to the electrolyte itself, other than forming a stable interphase, it is essential to find suitable electrolytes with a decreased freezing point as well as high conductivity to lower the ohmic polarization.

**Figure 2:**
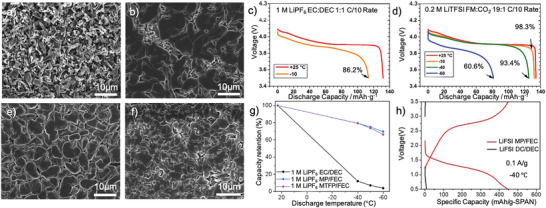
a,b) SEM images of plated Li metal morphology in 1.2 m LiPF_6_‐EC/EMC and 1 m LiFSI‐DME. a,b) Reproduced with permission.^[^ ^35^ ^]^ Copyright 2017, American Chemical Society. c,d) Voltage versus discharge capacity over various temperatures at the C/10 rate, using EC/DEC and FM/CO_2_ based electrolyte. Reproduced with permission.^[^ ^39^ ^]^ Copyright 2020, Elsevier. e,f) SEM images of plated Li metal morphology in 1.2 m LiTFSI‐ACN‐FM at room temperature and 1.2 m LiTFSI‐ACN‐FM at −60 °C. Reproduced with permission.^[^ ^40^ ^]^ Copyright 2020, Royal Society of Chemistry. g) low temperature discharge behavior of EC/DEC, MP/FEC and MTFP/FEC electrolyte. Reproduced with permission.^[^ ^42^ ^]^ Copyright 2020, American Chemical Society. h) Li||SPAN half cells performance at ultra‐low temperature.Reproduced with permission.^[^ ^43^ ^]^ Copyright 2018, Royal Society of Chemistry.

On the other hand, the Li metal deposition morphology also impacts the cycling performance of LBs, which is determined by the Li‐ion desolvation and Li nucleation and growth process. Therefore, an optimized Li‐ion solvation structure and stable SEI structure is essential to realize the dense and uniform Li deposition, especially under low temperature. In carbonate‐based electrolyte, the Li metal tend to form needle‐like dendrites (**Figure** **2**a) with a non‐uniform distribution, which leads to a porous bulk structure with high tortuosity, leading to low CE over cycling. A dendrite‐free deposited Li with large roundly shaped Li particles was enabled by the ether‐based electrolyte, indicating its homogenized Li deposition (Figure 2b). Clearly, ether‐based electrolyte demonstrates the highest ionic conductivity of 0.4 mS cm^−1^ at −80 °C and the smallest overpotential below 0.4 V during stripping/plating process, indicating its favored kinetics. Besides, the LiF‐rich SEI formed at lower temperature was found to be thinner, chemically and structurally distinct, and less resistive in comparison to the SEI formed at room temperature, indicating its admirable stability.^[^ ^35^ ^]^ However, a porous Li layer morphology with much lower and inconsistent CE can still be observed when cycled LMA in ether‐based electrolyte at −40 °C. This is likely due to the non‐even stripping/plating process on LMA owing to the small particle‐like Li deposition. Thus, the sluggish Li‐ion desolvation process could be the most critical issues that impedes the Li‐ion transportation and deposition on LMA, rendering unsatisfied low temperature performance.

### 3.1 Co‐Solvent

#### 3.1.1 Co‐Solvent in LMBs

The sluggish Li‐ion transportation and desolvation kinetics of high *T*_m_ EC solvent in EC/DMC electrolyte has been the primary blame for the poor performances. Initially, low *T*_m_ carbonate solvent including EMC was introduced as co‐solvent to improve the ionic conductivity and electrochemical stability of electrolyte.^[^ ^36^ ^]^ A high discharge capacity of 127.2 mAh g^−1^ under −20 °C can be observed in Li|LiCoO_2_ batteries by using EC/DEC/EMC based electrolyte.^[^ ^36b^ ^]^ However, the sudden ionic conductivity drop below −20 °C induces impeded ion movement in electrolyte, leading to unsatisfied performance. In fact, only 52% of discharge capacity can be retained under −40 °C.^[^ ^36b^ ^]^ The enhanced charge transfer kinetics on the LMA interface was further realized by employing FEC as co‐solvent. The FEC/EC/PC/EMC based electrolyte exhibits high ionic conductivity of 1.8 mS cm^−1^ under −40 °C and reduced charge transfer resistance, confirming its favored interfacial charge transportation.^[^ ^37^ ^]^ However, the Li|NMC cell could only yield 51% of its room temperature capacity, due to the electrolyte viscosity increase with a large portion of FEC. For extreme low temperature operation below −40 °C, difluoromethane (FM) and carbon dioxide (CO_2_) were used in formulating electrolyte.^[^ ^38^ ^]^ At −10 °C, the FM/CO_2_ based electrolytes show a 98.3% discharge capacity retention relative to 25 °C, which exceed the capacity retention of EC/DEC based electrolyte (86.2%) (Figure 2c,d). Even under −60 °C, the cell still exhibits high capacity retention of 60.6%, at which traditional liquid electrolytes would generally freeze.^[^ ^38^ ^]^ Using lower *T*_m_ and lower viscosity co‐solvent has been recognized as the most favorable approach so far adopted by the researchers with the aim to develop an electrolyte for low temperature applications. Thus, introducing THF as co‐solvent in FM/CO_2_ electrolyte facilitates cations coordination, which greatly enhances salt dissociation and transportation, rendering high Li^+^ transference number and improved CE of LMA. A much enhanced cyclic stability and high CE can be observed when cycled LMA in THF/FM/CO_2_ electrolyte.^[^ ^39^ ^]^ Even under extremely low temperature of −60 °C, a higher average CE of 98.4% can be achieved comparing with carbonate‐ and ether‐ based electrolyte, indicating the high electrochemical reversibility of LMA during stripping/plating process. Acetonitrile (ACN) was further used as co‐solvent in FM to further facilitate desolvation kinetics and reduce the operation temperature, which delivers a remarkable conductivity above 4 mS cm^−1^ even under −78 °C.^[^ ^40^ ^]^ The Li deposited in the liquefied gas electrolyte demonstrated a roundly shaped, densely packed dendrite free surface at both room temperature and extremely low temperature (Figure 2e,f). This indicates that the Li growth during deposition is completely uniform, which is synchronously improved by the high desolvation kinetics of the electrolyte and the stable interface of SEI. As a consequence, superior capacity of 65% can be observed when discharged at −60 °C, indicating its stupendous low temperature performance. However, the gas type electrolyte can only be liquefied under high pressure, which put forward a strict requirement for battery housing design. Once the electrolyte leaks, the release of environmental hazardous FM will be a crucial problem. Therefore, Smart et al.^[^ ^41^ ^]^ reported the all‐carbonate based electrolyte formulations by incorporating low melting, low viscosity linear acetate as co‐solvents to further improve the low temperature conductivity down to −70 °C. To examine its performance in LBs, John et al.^[^ ^42^ ^]^ introduced methyl propionate (MP) or methyl 3,3,3‐trifluoropionate (MTFP) as co‐solvent with FEC for LBs to realize improved reaction kinetics (Figure 2g). Employing 1 m LiPF_6_ in MTFP/FEC (9:1 volume ratio) yielded an excellent capacity retention of 80% for Li|LiNi_0.8_Co_0.1_Mn_0.1_O_2_ batteries charged to 4.5 V after 250 cycles. Attributed to its solvation structure superiorities, the Li||sulfurized polyacrylonitrile (SPAN) cell with LiFSI MP/FEC electrolyte exhibited appealing Li metal compatibility than LiPF_6_ EC/DEC electrolyte (CE: 94.2% vs 88.3%, room temperature) and 78% capacity retention at −40 °C, corresponding to an admirable cyclic stability with a low capacity fading rate of 0.086% per cycle.^[^ ^43^ ^]^

#### 3.1.2 Co‐Solvent in Li‐Chalcogenide Batteries

As for ether electrolyte based Li‐S batteries, Ryu et al.^[^ ^44^ ^]^ introduced MP into TEGDME‐DOL based electrolyte to endow a stable Li stripping/plating process in Li‐S batteries under low temperature. Attributed to this advantage, the cell displayed a high initial discharge capacity of 1342 mA h g^−1^ at 20 °C and a decent capacity retained close to 1000 mA h g^−1^ at −10 °C, which is three times higher than that in TEGDME based electrolyte (Figure 2h). Recently, Holoubek et al.^[^ ^45^ ^]^ developed a new ether‐based electrolyte by employing diethyl ether (DEE) as solvent and LiTFSI as solute. The promoted desolvation kinetics of DEE enables the Li‐S batteries with an excellent performance when paired with high‐loading (3.5 mAh cm^−2^) SPAN cathode with a onefold excess LMA. The cell can still retain 84% and 76% of its room temperature capacity when cycled at −40 and −60 °C, respectively.

The electrolyte component design becomes the most critical issues for Li‐O_2_ batteries since the commonly used high *T*_b_ solvent is able to freeze at low temperatures, which results in sluggish redox reaction kinetics and hinders its practical application. The discharge capacity of Li‐O_2_ cells and the morphology of Li_2_O_2_ are significantly governed by the environmental temperature, which decreases slowly from 7492 mAh g^−1^ at 40 °C to 2930 mAh g^−1^ at 0 °C, but further increases sharply to an extraordinarily high capacity of 17716 mAh g^−1^ at −20 °C.^[^ ^46^ ^]^ A much extended cycle life can be observed in Li‐O_2_ batteries when operating at 0 °C, indicating its superior low‐temperature cycling stability.^[^ ^47^ ^]^ This low temperature performance invigoration could be related to the inhibited electrolyte decomposition and prolonged lifetime of superoxide at low temperature, enabling the anion as a redox mediator and contributing to a stable interface formation.

### 3.2 Cation Solvation Structure

#### 3.2.1 Cation Solvation Structure in LMBs

**Figure 3:**
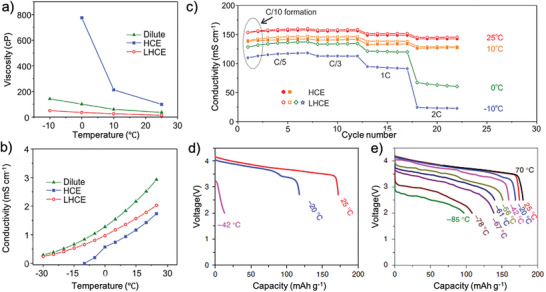
a) Viscosity and b) ionic conductivity of sulfone‐based electrolytes under different temperatures; c) comparison of discharge rate capabilities of LiNi_1/3_Co_1/3_Mn_1/3_O_2_/Li batteries in HCE and LHCE at different temperatures. a–c) Reproduced with permission.^[^ ^48^ ^]^ Copyright 2018, Elsevier. d) discharge profiles of LiNi_1/3_Co_1/3_Al_1/3_O_2_/Li batteries using conventional 1 m LiPF_6_‐EC/DMC electrolyte at different temperatures; e) discharge profiles of LiNi_1/3_Co_1/3_Al_1/3_O_2_/Li batteries using 1.28 m LiFSI‐FEC/FEMC‐D2 electrolyte at different temperatures. Reproduced with permission.^[^ ^50^ ^]^ Copyright 2012, Springer Nature.

Optimizing cation solvation structure is also capable of changing the desolvation capability of Li^+^ to facilitate Li intercalation or deposition, which improves its reaction kinetics and realizes a good rate performance under low temperature. Although increasing Li salt concentration beyond 1 m is capable of offering more free Li^+^ in the electrolyte and increase ionic conductivity, the high concentration electrolyte (HCE) is unable to realize large scale application since the large amount of Li salt used in electrolyte is not a cost‐effective strategy. Recently, local high concentration electrolyte (LHCE) was developed to reduce the electrolyte polarity and increase Li‐ion transference number. The utilization of LHCE increases the liquid temperature range and reduce the Li salt content in electrolyte, given rise to a much‐improved electrochemical performance for wide‐service temperature. Comparing with HCE, the good process capability in present cell manufacturing can be realized owing to its low viscosity and good wettability. Ren et al.^[^ ^48^ ^]^ compared the ionic conductivity and electrolyte viscosity of HCE and LHCE in sulfonate based electrolyte under low temperature (**Figure** **3**a,b). The exponentially increased viscosity below 10 °C coupling with fast ionic conductivity drop in HCE impede the charge transfer in LBs. On the other hand, only slight change of viscosity and conductivity can be observed in LHCE with the temperature decreasing. Although the LiNi_1/3_Co_1/3_Mn_1/3_O_2_/Li batteries using the HCE fails to operate at 0 °C, the LHCE can still retain a large fraction of cell capacity up to 2 C under the low temperature of −10 °C (Figure 3c). To further enhance the stability of LMA, Dong et al.^[^ ^49^ ^]^ introduced dichloromethane (DCM) as diluent into concentrated ethyl acetate (EA) based electrolyte, which shows high ionic conductivity of 0.6 mS cm^−1^ and low viscosity of 0.35 Pa⋅s with a wide potential window at −70 °C. When pairing with polyimide (PI) as cathode material, the Li||PI cells delivered a high discharge capacity of 84 mAh g^−1^ and decent capacity retention of 83.5% over 100 cycles under current density of 0.2 C. All‐fluorinated, non‐flammable electrolytes have high ionic conductivity and a wide electrochemical stability window. However, LBs using all‐fluorinated electrolytes cannot work at temperatures below −30 °C due to the high affinities between the fluorinated solvents and the Li ions. Therefore, reducing the affinities between the Li^+^ and solvents by introducing highly fluorinated non‐polar solvents is the best tactic. Fan et al.^[^ ^50^ ^]^ reported a novel strategy to introduce non‐polar tetrafluoro1‐(2,2,2‐trifluoroethoxy)ethane (D2) as diluent into FEC and methyl (2,2,2‐trifluoroethyl) carbonate (FEMC) to formulate electrolyte, thus achieving an all‐fluorinated, non‐flammable LHCE with high ionic conductivity and superior electrochemical stability under extremely low temperature. When the temperature was reduced to −42 °C, the LiNi_1/3_Co_1/3_Al_1/3_O_2_/Li batteries in 1.28 m LiFSI‐FEC/FEMC‐D2 electrolyte could still provide a high capacity of 160 mAh g^−1^, while the LiNi_1/3_Co_1/3_Al_1/3_O_2_/Li batteries in the 1 m LiPF_6_ carbonate‐based electrolyte only provided a capacity of 13.3 mAh g^−1^ due to the electrolyte solidification below −30 °C (Figure 3d,e). This cell could still deliver a decent capacity of 96 mAh g^−1^ under extremely low temperature of −85 °C, indicating its superior low temperature electrochemical performance.

#### 3.2.2 Cation Solvation Structure in Li‐Chalcogenide Batteries

HCE is also promising to improve Li‐S performance since the large amount of Li salt in electrolyte inhibits the dissolution and dissociation of LiPS, leading to suppressed polysulfide shuttle effect. Suo et al.^[^ ^51^ ^]^ developed the “solvent‐in‐salt” ultrahigh concentration electrolyte (7 m LiTFSI in DOL/DME solvent) for Li‐S batteries, which provided a high lithium‐ion transference number of 0.73 and high conductivity of 0.814 mS cm^−1^. Attributed to the electrolyte advantages, a satisfied rate performance and good cyclic stability of Li‐S performance could be achieved under −20 °C. To further reduce the solvent decomposition on LMA, a solidified electrolyte was also developed in Li‐O_2_ batteries, which delivered a remarkable performance under extremely low temperature of −73 °C.^[^ ^52^ ^]^

### 3.3 Anion Modification of Li Salts

#### 3.3.1 Li Salts in LMBs

Although LHCE seems to be the promising solution for wide application under low temperature, the introduction of large portion of non‐polar diluent that unable to dissociate Li salt can also decrease the ionic conductivity of electrolyte, endowing battery with sluggish reaction kinetics and unsatisfied performance. The anion modification of Li salts with favored dissociation capability is able to offer more Li^+^ in electrolyte and enhance its ion mobility, leading to enhanced redox reaction kinetics. Besides, the preferential adsorption and reduction of anions on LMA constructs stable SEI for long life LBs by altering its anion structure.

**Figure 4:**
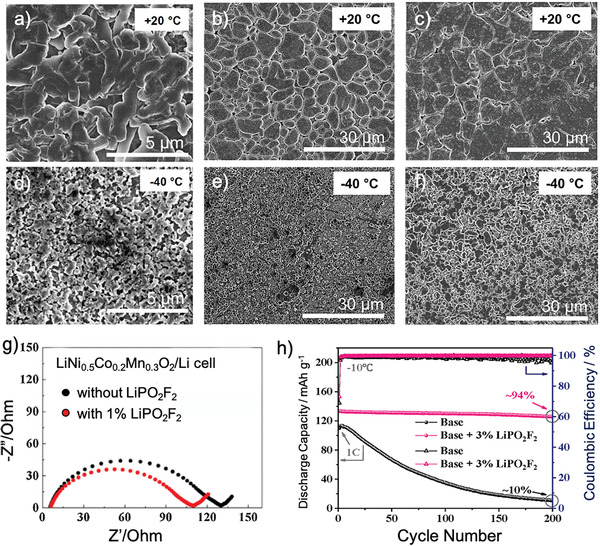
a–f) Li metal deposition morphology in (left) DOL/DME based electrolyte, (middle) with LiNO_3_ additive and (right) with LiNO_3_‐FEC additives under 20 and −40 °C. a,d) Reproduced with permission.^[^ ^35^ ^]^ Copyright 2017, American Chemical Society. d,b,c,e,f) Reproduced with permission.^[^ ^15a^ ^]^ Copyright 2020, American Chemical Society. g) EIS spectra of LiNi_0.5_Co_0.2_Mn_0.3_O_2_/Li batteries under 0 °C; h) discharge profiles of LiNi_1/3_Co_1/3_Mn_1/3_O_2_/Li batteries at −10 °C, containing LiPO_2_F_2_ as electrolyte additive. g,h) Reproduced with permission.^[^ ^67^ ^]^ Copyright 2018, Elsevier.

Comparing with LiPF_6_ based carbonate electrolyte, LiBF_4_,^[^ ^53^ ^]^ LiAsF_6_,^[^ ^54^ ^]^ and Lithium oxalyldifluoroborate (LiDFOB)^[^ ^55^ ^]^ based carbonate electrolyte offer higher ion mobility for improved rate performance in LBs. In 2006, Zhang et al.^[^ ^56^ ^]^ first investigated the low temperature performance of different Li salt in Li||LiFePO_4_ cells. Although LiBF_4_ based electrolyte exhibits lower ionic conductivity than LiPF_6_ based electrolyte, the lower charge transfer resistance achieved with tetrafluoroborate anions leads to better capacity retention at −30 °C.^[^ ^57^ ^]^ Therefore, LiBF_4_ based binary salts were employed in carbonate electrolyte and the performance with different ratio was evaluated, as shown in **Figure** **4**a–f. The LiBF_4_–LiBOB binary system with a small amount of LiBOB exhibited the highest discharge capacity at −30 °C.^[^ ^56^, ^58^ ^]^ To further accelerate reaction kinetics, LiDFOB, which has the combined structural advantages of LiBOB and LiBF_4_, was developed as Li salt, resulting in better low temperature rate performance and ≈68% of discharge capacity at −30 °C.^[^ ^55^ ^]^ In order to inhibit dendrite growth of LMA, two new electrolytes are formed by using boron‐based anion receptors, tris(pentafluorophenyl) borane (TPFPB), or tris(2*H*‐hexafluoroisopropyl) borate (THFPB) as additives to dissolve the LiF salt in carbonate solvents. When employed in LiMn_2_O_4_/Li batteries, the cell not only demonstrates significantly improved dendrite growth inhibition, but also acquire a high ionic conductivity of 1 mS cm^−1^ under −40 °C and high Li^+^ transference number of 0.7.^[^ ^59^ ^]^

#### 3.3.2 Li Salts in Li‐Chalcogenide Batteries

The success of anion structure manipulation also inspires the Li salt design in Li‐S batteries. It was reported that employing lithium triflate (LiTf) in DOL/DME solvent as electrolyte could enhance the low temperature Li‐S performance.^[^ ^60^ ^]^ Even under high current density of 5 C, a 60% of room temperature discharge capacity could still be obtained under −40 °C, holding great promises in promoting the practical application of low temperature Li‐S batteries.^[^ ^61^ ^]^

### 3.4 Electrolyte Additives

**Figure 5:**
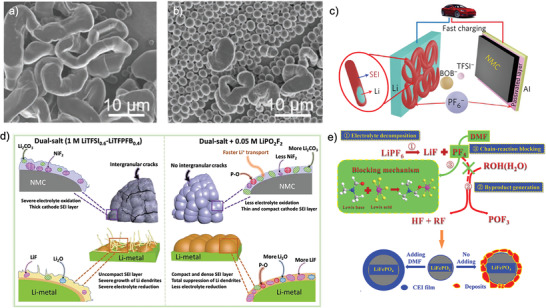
SEM images for the Li plating morphology on copper substrates under a) 60 °C and b) 25 °C with the current density of 0.5 mA cm^−2^ and 0.5 mAh cm^−2^. a,b) Reproduced with permission.^[^ ^75^ ^]^ Copyright 2019, Wiley‐VCH. c) schematic illustration demonstrating that a LiPF_6_ additive in LiTFSI–LiBOB electrolyte prevents the Al corrosion and improves the stability of Li metal. Reproduced with permission.^[^ ^62^ ^]^ Copyright 2019, Springer Nature. d) schematic illustration showing the positive effects of LiPO_2_F_2_ additive on interfaces of both Li‐metal anode and NMC cathode during repeated cycling in dual‐salt electrolyte, at 60 °C. e) blocking mechanism of DMF for decomposition of LiPF_6_, the generation of by‐products, and dissolution of LiFePO_4_ electrode during charge and discharge. d,e) Reproduced with permission.^[^ ^91^ ^]^ Copyright 2019, American Chemical Society.

The sharp decline of cell behavior at subzero temperatures is the combined consequence of the decreased capacity utilization and depressed cell potential, and this performance deterioration can be attributed to the increased resistance of the SEI and the resistance associated with charge‐transfer processes at both cathode and anode interfaces. Employing electrolyte additives serves as a promising proposition for performance enhancement owing to its capability to alter the composition and structure during SEI formation. The introduction of electrolyte additive facilitates the formation of stable SEI on LMA, which regulates uniform Li deposition and inhibits Li dendrite growth. The additive can also endow LBs with multifunctional properties to achieve a fast and durable performance. In DOL/DME based electrolyte, the Li metal deposition morphology exhibits much smaller particle sizes and severe dendrite growth at lower temperatures, leading to lower average CE over cycling.^[^ ^35^ ^]^ The introduction of LiNO_3_ regulates the deposition layer from porous loosen morphology into dense structure, leading to improved CE under low temperature.^[^ ^15a^ ^]^ Interestingly, at lower temperatures (20 °C and −40 °C), the FEC contained electrolyte features the largest average particle size and the most compact and uniform films. This result indicates that the fluoride additive facilitates the formation of densified Li deposition, conferring stable SEI for enhanced interfacial stability. Based on this observation, LiPF_6_ was further employed as additive for improved low temperature performance, which can also inhibit current collector corrosion on the cathode.^[^ ^62^ ^]^ With a tiny amount of LiPF_6_ additive, a significantly enhanced charging capability and cycling stability of LBs can be realized when cycled in LiTFSI–LiBOB dual‐salt/carbonate‐solvent‐based electrolytes. The success implantation of electrolyte additive for enhanced LMA performance inspired researchers to investigate the low temperature performance enhancement by additives. Ota et al.^[^ ^63^ ^]^ first revealed that the Li metal deposition morphology and cyclic stability were improved by vinylene carbonate (VC) additive. In EC/DMC based electrolyte, numerous needle‐like dendritic Li can be seen in the loosen deposition layer, indicating its Li dendrite formation. After introducing VC, a denser Li deposition layer could be observed, contributing to the improved capacity retention. Adding butyl sultone (BS) into electrolyte also participated the formation of thin SEI film, which could enhance the ion conductivity of the SEI film and accelerate the Li^+^ migration through the SEI film.^[^ ^64^ ^]^ Ethoxy(pentafluoro) cyclotriphosphazene (PFPN) also employed as a multifunctional flame retardant additive to confer a non‐flammable electrolyte, which also produced a more stable dense SEI for improved rate performance at −20 °C.^[^ ^65^ ^]^ The lithium difluorophosphate (LiPO_2_F_2_) electrolyte additive significantly improves the charge transfer kinetics under low temperature, as revealed by the EIS spectra of LiNi_1/3_Co_1/3_Mn_1/3_O_2_/Li batteries (**Figure** **5**B).^[^ ^66^ ^]^ Electrochemical performance of high voltage LiNi_1/3_Co_1/3_Mn_1/3_O_2_/Li batteries presented a good capacity retention of 94% after 200 cycles at −10 °C (Figure 4g). Even at a high current density of 10 C, the cell still delivered a high initial capacity of 144 mAh g^−1^ and maintains at 69 mAh g^−1^ after 1000 cycles at room temperature.^[^ ^67^ ^]^ The improved cyclic stability and rate capability of high voltage LiNi_0.5_Co_0.2_Mn_0.3_O_2_ were mainly ascribed to the steady low impedance CEI film created by 3% LiPO_2_F_2_ additive, which greatly hindered the subsequent electrolyte oxidation, electrode structural destruction and increase of electrode polarization during cycling (Figure 4h). Jones et al.^[^ ^68^ ^]^ further examined the polarization resistance on the cathode with different types of Li salts additives. The electrolyte with LiBOB additive produced the lowest polarization resistance at −40 °C (2.42 Ω), indicating the best charge transfer kinetics. Borate based additive including tris(pentafluorophenyl) borane was also able to induce stable SEI formation, giving rise to a much smaller interfacial resistance for low temperature operation. In addition, solid‐state insoluble additives such as PMMA could also reinforce SEI layers and inhibit electrolyte frozen, leading to enhanced charge transfer kinetics on LMA under low temperature.^[^ ^69^ ^]^ Overall, the implementation of additive contributes to the stable interface formation, leading to reduced charge transfer resistance on the interface for enhanced rate capability and extended cyclic stability for prolonged lifespan.

### 3.5 Solid‐State Electrolyte

Solid State Electrolytes (SSEs) are milestones in the technology roadmaps for safe and high energy density batteries. However, the Li^+^ movement in SSE need to overcome much higher energy barrier comparing with that in liquid electrolyte. The slow ion transportation in SSE results in sluggish charge transfer kinetics, rendering limited ionic conductivity and severe polarization. To deal with this conundrum, polymer‐based SSE is designed to enhance charge transportation for low temperature operation. Xu et al.^[^ ^70^ ^]^ introduced succinonitrile into polyethylene oxide (PEO)‐based SSE for Li|LFP cells to suppress the PEO crystallization and mitigate the affinity between EO and Li^+^, giving rise to a decent ionic conductivity of 2 × 10^−5^ S cm^−1^ at 0 °C. The formed homogeneous PEO‐based SSE with fast Li^+^ transport channels enabled the cell with a high discharge capacity of 118.6 mAh g^−1^ over 180 cycles. Comparing with polymer‐based SSEs, inorganic SSEs have been deemed as the potential candidate of SSEs for low temperature operation, which have received extensive concern as they can integrate the advantages and avoid the disadvantages of inorganic and organic electrolytes. Among inorganic SSEs, metal organic frameworks (MOFs) exhibit significant advantages for low temperature operation, thus effectively eliminate the safety concerns of LMBs. Zhang et al.^[^ ^71^ ^]^ demonstrated that employing UIO‐66 based MOFs as SSEs could acquire an ionic conductivity of 6.7 × 10^−5^ S cm^−1^ at −20 °C. When utilizing this SSE in Li|LFP cells, a stable cycling performance at 0.05 C can be obtained. However, the poor ionic conductivity and undesired Li dendrite growth impede its wide‐scale application. To solve this problem, single‐ion conductor was developed to facilitate Li^+^ transportation and inhibits Li dendrite growth. A UIO‐LiTFSI based single‐ion conductor was developed and serve as low temperature SSE for LMB. It demonstrated high rate performance and decent discharge capacity (56 mA h g^−1^ under the current density of 2 C) at 0 °C in Li|LFP cells.^[^ ^72^ ^]^ A single‐ion conductive covalent organic frameworks (COFs) based SSE was also designed, which exhibited high Li^+^ transference number of 0.92 at 20 °C, and a high ion conductivity of 10^−5^ S cm^−1^ that can be sustained down to low temperature of −40 °C. When paired with quinone‐based organic molecules as cathode, an impressive cycle stability could be obtained.^[^ ^73^ ^]^

## 4 Strategies for High Temperature Electrolyte Design

Under high temperature, the ionic conductivity of electrolyte will no longer impedes the ion migration. High CE and dendrite‐free morphologies of LMA can be achieved under thermodynamics and kinetics favored deposition conditions. However, LMBs should operate no higher than the melting point of Li metal (≈180 °C) due to the substantially increase reaction kinetics of electrolyte with Li and it is considered as upper limit for temperature selection. Throughout literature, the volume of works focused on elevated temperature (<180 °C but higher than room temperature) LMBs is scarce. Most papers of this nature are focused on the enhanced ionic conductivity of SSE and the processes occurring at the anode are not appreciably studied. Nevertheless, various surprising and interesting phenomena can occur at elevated temperature. For example thicker and more stable SEI layer can be formed on the Li anode surface at higher temperatures (due to the increase electrolyte decomposition kinetics), which could suppress electrolyte decomposition, prevent dendrite formation and inhibit polysulfide corrosion.^[^ ^74^ ^]^ In addition, the Li nucleation/growth process is highly dependent on the temperature and the Li morphology and deposition structure can be altered under high temperature. The Gibbs energy change ΔG for the Li nucleation process can be described by the following equations:

(2) $$\Delta G=\frac{16\;\pi \;\gamma^{3}V_{m}^{2}\Phi }{3z^{2}e^{2}\eta^{2}}$$

where *γ* relates to the surface tension between nuclei and electrolyte, *V*_m_ is the molar volume of Li, Φ reflects the nucleation activity of the substrate, z is the valence of plating Li ion, e is the elementary charge, *η* is the nucleation overpotential, and F is the Faraday's constant. Under higher temperature, a smaller *γ* can be observed and a smaller *η* is required for Li nucleation while bigger Li nuclei is required for plating process. Thus, a much bigger Li plating structure with reduced surface area can be observed under high temperature, inhibiting side reaction and high CE over cycling (Figure 5a,b).^[^ ^75^ ^]^

However, cycling tests of LBs above 60 °C have been rarely reported in the literature, most likely owing to the chemical instability of Li salt, especially LiPF_6_ in the organic solvents at elevated temperature. While more stable SEIs could be formed at higher temperatures, severe Li oxidation and SEI accumulation also can occur under high temperature, which continuously consumes electrolyte and Li, leading to sluggish ion transportation low CE over cycling.^[^ ^76^ ^]^ The specific balance between these two very polar processes is unclear. Furthermore, violent reaction will occur once the highly reductive Li contact with highly oxidative Li salt that precipitated during solvent vaporization, rendering battery explosion. On the cathode side, the hydrolysis of fluoride Li salt induce the HF generation, which results in metal ion dissolution of cathode material and current collector corrosion in LMBs, leading to fast capacity fading. As for conversion‐type LBs, a much lower activation energy can be observed under higher temperature, which results in smaller overpotential during charge‐discharge process, leading to favored charge transfer kinetics for accelerated redox reaction.^[^ ^77^ ^]^ Compared with that at ambient temperature, the performances of discharging and charging have been improved at 70 °C since the discharging capacity increased to about 80% and the charging voltage plateau decreased from 4.2 to 3.5 V.^[^ ^78^ ^]^ Operating under elevated temperature is more challenging in Li‐S batteries since the ether based electrolyte does not enable a safe operation due to the low *T*_b_ and flash temperatures. However, the carbonate‐based electrolyte Li‐S batteries demonstrate severe side reactions with LiPS intermediates, resulting in irreversible electrochemical behavior. In order to design high temperature electrolytes with inhibited side reaction, the solvents should possess a combination of several critical properties, such as high dielectric constant, low viscosity, adequate coordination behavior, as well as appropriate liquid ranges and salt solubility in the medium. However, the high dielectric constant of the solvents inevitably enhances the dipole‐dipole force among these highly polar molecules, increasing the freezing temperature of the solvents and thus reducing the low‐temperature performance of the electrolytes. With respect to the electrolyte itself, other than forming a stable interphase, the greatest concern is to find suitable electrolytes with a decreased freezing point as well as high conductivity to lower the ohmic polarization.

### 4.1 Solvent Component

The independent and incomplete decomposition of Li salts is enhanced at high working temperature. The intermediate products, such as LiN*_x_*O*_y_* and products with S‐F bond generate at elevated temperature. Moreover, high temperature accelerates the decomposition process of NO_3_^−^, weakening the synergistic effect of NO_3_^−^ and FSI^−^, which promotes specific spatial distribution of inorganic components in SEI. On the other hand, the incomplete disintegration of FEC solvent is enhanced at 90 °C, forming more organic components (C‐F species) instead of LiF. Less LiF with high surface energy induces a high interfacial impendence and non‐uniformity of SEI at 90 °C comparing with that at 25 °C. Consequently, a relatively nonuniform Li utilization can be observed at elevated temperature.^[^ ^79^ ^]^ LHCE exhibits higher ionic conductivity and electrochemical performance under raised temperature. However, the severe safety concern significantly impedes its practical application. The slow electrolyte decomposition and SEI formation induce the continuously loss of solvent, which further increases the Li salt concentration and induce precipitation once oversaturation. The fast exothermic reaction between precipitated oxidative Li salt and highly reductive LMA may results in battery fire or explosion. To achieve better performance under higher temperature, SSEs should be introduced. An intrinsic flame‐retardant (IFR) electrolyte is presented consisting of 1.1 m LiFSI in a solvent mixture of flame‐retardant TEP and high flashpoint solvent 1,1,2,2‐tetrafluoroethyl‐2,2,3,3‐tetrafluoropropyl ether (TTE) for high temperature Li‐S performance up to 60 °C.^[^ ^80^ ^]^

### 4.2 Li Salts

Severe LMA and cathode material corrosion will occur in LBs under raised temperature owing to POF_3_ and HF generation via LiPF_6_ decomposition. In order to improve thermal stability, dual salts were employed in the electrolyte for longer cycle life, which is able to alter the interfacial chemistries and introduce entirely new interphases via preferential decomposition on the LMA, rendering a dense and conformal Li deposition morphology.^[^ ^81^ ^]^ To scavenge water moisture in electrolyte and reduce LiPF_6_ hydrolysis as well metal dissolution of LMBs under high temperature, lithium 2‐trifluoromethyl‐4,5 dicyanoimidazole (LiTDI) has been introduced with LiPF_6_ as dual salts for enhanced thermal stability.^[^ ^82^ ^]^ However, LiTDI based electrolytes demonstrates high irreversible capacities and low CE attributed to its poor film‐forming capabilities.^[^ ^83^ ^]^ Dilithium dodecafluorododecaborate (Li_2_B_12_F_12_) was further introduced with LiPF_6_ as dual salts in carbonate electrolyte, which delivers a relatively higher capacity retention over 60% at 60 °C.^[^ ^84^ ^]^ To improve the cycling stability, borate based dual salts were employed in the electrolyte. Jiao et al.^[^ ^85^ ^]^ discovered that the combination of LiDFOB and LiTFSI could introduce abundant electron‐deficient B atoms to coordinate with other electron rich anions, leading to the formation of the polymeric SEI layer and the improved the quality of the anode SEI layer for better Li metal protection. Besides, Chen et al.^[^ ^86^ ^]^ reported the lithium amide–lithium borate (such as LiFSI–LiDFOB, LiTFSI–LiDFOB, and LiTFSI–LiBOB) dual‐salt electrolytes, which enabled excellent cycling performance up to 60 °C. On one hand, the LiPF_6_ additive is a critical piece in stabilizing Al foil and maintaining electrical connection with the active material. On the other hand, a small amount of additive greatly alters the nature of the SEI layer on LMA. The SEI layer produced in LiPF_6_‐added dual‐salt electrolyte is highly conductive and has very limited effects on the electrode polarization, which could prevent the accumulation of isolated/‘dead’ Li during each deposition/stripping cycle. In addition, the polycarbonates formed in the SEI layer are flexible, which can efficiently cover the Li metal surface, reduce the side reactions, hold the isolated/"dead" Li particles tightly and adhere to the bulk Li anode, thus preventing the detachment of the SEI layer from the bulk Li metal (Figure 5c). Zhang et al.^[^ ^87^ ^]^ further investigated the LiBF_4_‐LiDFOB carbonate based electrolyte for LiCoO_2_/Li batteries. A reduced charge transfer resistance and enhanced cyclic stability could be observed in electrolyte with 0.2 m LiDFOB and 0.8 m LiBF_4_. Attributed to the remarkable interfacial stability, an apparently high capacity retention of 93.5% could be achieved over 100 cycles under 60 °C. Borate based salts were also employed in Li‐S batteries in an attempt to enable a high performance under raised temperature. Yang et al.^[^ ^88^ ^]^ designed the 1 m LiBOB triethyl phosphate (TEP)/FEC electrolyte, which enabled the S cathode with satisfactory capacity retention of 91.3% after 500 cycles at 1 C.

### 4.3 Electrolyte Additives

#### 4.3.1 Electrolyte Additives in LMBs

The success of introducing lithium borate as electrolyte salts further inspires the scientific community to discover the capability of lithium borate as electrolyte additive to regulate lithium deposition morphology. The surface morphologies of LMA after cycling shows that LiBOB additive enables the formation of smooth and dense surface, leading to stable stripping/plating process over long‐term operation.^[^ ^89^ ^]^ To further enable a long‐term operation of LMBs, LiPO_2_F_2_ was employed as additive in borate based dual salts electrolyte, which afforded a very dense and compact morphology without significant cracks and any Li dendrites, indicating its sufficient protection of bulk fresh Li at 60 °C. Figure 5d illustrates the synergistic effects of LiPO_2_F_2_ additive on interfaces of both LMA and NMC cathode. On the one hand, LiPO_2_F_2_ assisted enrichment of inorganic lithium compound to form compact SEI layer, which suppressed electrolyte reduction and Li dendrites growth. On the other hand, LiPO_2_F_2_ assisted enrichment of inorganic lithium species at cathode surface, which prevented structural degradation and electrolyte oxidation.^[^ ^90^ ^]^ Attributed to these features, the LiNi_1/3_Co_1/3_Mn_1/3_O_2_/Li batteries showed significantly improved discharge capacity retention of 69.6% and high average CE of 99.6% over 300 cycles. Meanwhile, numerous Lewis base molecules, such as DMF,^[^ ^91^ ^]^ 2,3,4,5,6‐pentafluorophenyl methanesulfonate (PFPMS),^[^ ^92^ ^]^ VC,^[^ ^93^ ^]^ triphenyl borate (TPB),^[^ ^94^ ^]^ trimethyl borate (TMB)^[^ ^94^ ^]^ trimethylsilylcyclopentadiene (SE),^[^ ^95^ ^]^ propane sultone (PS)^[^ ^96^ ^]^ and 2‐(triphenylphosphoranylidene) succinic anhydride (TPSA)^[^ ^97^ ^]^ were developed as electrolyte additives. These molecules with lone electron pair offer strong Lewis acid‐base interaction to capture Lewis acid in electrolyte, leading to inhibited side reaction. It is reported that the addition of DMF into LiPF_6_ based electrolyte could rapidly capture the Lewis acid PF_5_ generated from LiPF_6_ decomposition, therefore effectively blocking the further occurrence of side reactions and the corrosion of electrode materials (Figure 5e).

#### 4.3.2 Electrolyte Additives in Li‐Chalcogenide Batteries

As for Li‐S batteries, LiNO_3_ is the most commonly used additive owing to its excellent film forming capability on the synergism with LiPS as well as superior LiPS shuttle inhibition at room temperature.^[^ ^98^ ^]^ The as‐formed stable LiNO_3_/LiPS derived SEI layer can further endure the LiPS corrosion to realize a fast and durable Li‐S electrochemistry. However, since the side reaction is exponentially amplified with increasing temperature, adding 2 wt% LiNO_3_ additive to electrolyte is insufficient. Many literatures reported that a more stable cycling performance of Li‐S batteries can be acquired with 5% LiNO_3_ added in electrolyte, which might generate efficient Li*_x_*NO*_y_* from LiNO_3_ decomposition.^[^ ^99^ ^]^ The efficacy of Li_x_NO_y_ layer efficiently suppressed LiPS dissolution, migration as well as LMA corrosion, leading to high initial CE of 102.1% and stable operation over 400 cycles at elevated temperature of 60 °C.^[^ ^100^ ^]^

### 4.4 Solid‐State Electrolyte

In contrast to the other systems, the performance of SSE systems typically performs better at elevated temperature as their Li‐ion conductivity is significantly enhanced. Furthermore, as SSEs typically do contain relatively less organic solvent in their compositions, SSEs can be more thermally stable. With higher temperatures (<180 °C), their ionic conductivity increases resulting in enhanced performance. This section will highlight important design strategies for near‐solid‐state electrolyte systems and all‐solid‐state electrolyte at high temperatures and their disadvantage/advantages.

#### 4.4.1 Near‐Solid‐State‐Electrolytes

This class of electrolytes is composed of quasi‐solid state and gel‐type. Quasi‐solid state and gel‐type can be seen as the bridge between fully SSE and liquid electrolyte where minute amounts of liquid are used with solid material. Hybrid electrolyte can be viewed as a blend of polymer electrolyte and SSE particles. Accordingly, the properties of all these systems are also a compromise of liquid electrolyte and SSEs. Near‐solid electrolytes tend to be more mechanically flexible in comparison to true SSE systems, resulting in better contact with active materials. As mobility of molecules increases with temperature, the conductivity of near‐SSEs typically benefits quite substantially from elevated‐temperature (60 °C) operation.^[^ ^101^ ^]^ This makes this class of electrolytes possibly applicable for some moderate‐high temperature battery operation. However, they also tend to be more flammable than solid‐state systems due to the residual amount of solvent, which could be released at higher temperatures during cell failure.

Gel‐type electrolytes are mixtures of a polymer and liquid organic electrolyte. Common composition include PEO,^[^ ^102^ ^]^ polymethyl methacrylate (PMMA),^[^ ^103^ ^]^ poly(vinylidene fluoride‐co‐hexafluoropropylene) (PVDF‐HFP),^[^ ^104^ ^]^ among many others. The gel formed with two components has excellent contact with cell components due to its high mechanical flexibility. Its high temperature performance is mostly limited by its de‐gelling point. The glass transition point (*T*_g_) of a polymer electrolyte is key as it strongly correlates with the Li‐ion conductivity. A crystalline polymer network provides a large hindrance toward transport due to the rigidity of the polymer chains and the subsequent low mobility of Li‐ions.^[^ ^105^ ^]^ Use of plasticizer (common plasticizers^[^ ^106^ ^]^ used include ethylene carbonate, propylene carbonate, ethylene glycol, among many others organic molecules) significantly reduces the *T*_g_ to well‐below room temperature,^[^ ^106^ ^]^ allowing for Li‐ion transport. Quasi‐SSE has been loosely defined throughout literature but often includes use of solid particles (SiO_2_,^[^ ^107^ ^]^ Al_2_O_3_,^[^ ^108^ ^]^ Y_2_O_3_‐ZrO_2_,^[^ ^109^ ^]^ metal organic frameworks particles,^[^ ^110^ ^]^ SSE ceramics,^[^ ^111^ ^]^ among others) in combination with polymer and/or liquid organic electrolyte components (solvent and/or salt). Beyond the incorporation of solid particles, quasi‐SSE is very similar to gel‐type electrolytes.

It is expected that high temperature operation of near‐SSEs will be beneficial to the rate performance due to the increase conductivity. For example, the high temperature property of just a PVDF‐HFP membrane (prior to gelation with liquid electrolyte) was demonstrated to be quite exceptional with thermal stability of up to 350 °C and also was not easily ignited.^[^ ^112^ ^]^ However, upon uptake of liquid electrolyte (≈86 wt% liquid electrolyte), the stability decreased and the gel‐electrolyte loses up to 40 wt% of its mass at 150 °C, likely from liquid evaporation. Such a large release of liquid and high temperature mostly voids the safety benefits of using a near‐solid state system and brings back all the problems associated with use of liquid electrolytes. When the temperature is increased, the liquid component of the gel might destabilize (evaporate, leakage from gel), resulting in a significant decrease in performance (decrease conductivity, reactivity of free liquid electrolyte components with LMA) and also thermal runaways.^[^ ^113^ ^]^

Similarly, like gel‐type electrolytes, the need for an organic liquid component such as ethylene carbonate is almost always reported to serve the important role of a plasticizer for quasi‐SSE.^[^ ^114^ ^]^ Thermogravimetric analysis in N_2_ reveals a liquid electrolyte content of ≈45 wt% with the majority of the mass loss occurring at >180 °C. The proportion of electrolyte solvent in quasi‐solid systems are actually nearly identical to that of pure liquid electrolytes and the polymer/inorganic scaffold are often represented at additive levels or a few weight percent.^[^ ^115^ ^]^ While this temperature is likely below any LMA based battery system (very close to melting point of Li metal), significant leakage of the liquid organic electrolyte at high temperatures present problems for limiting thermal runaway.

**Figure 6:**
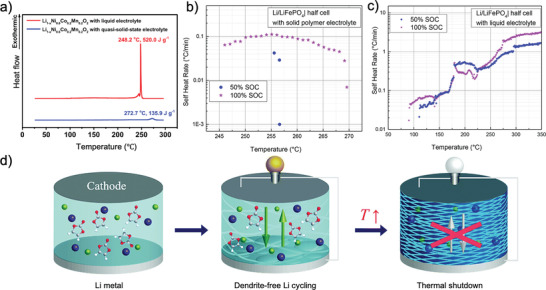
a) Differential scanning calorimetry of NMC 622 with liquid and quasi‐solid‐state electrolyte (LiTFSI, DMC, polycaprolactone triacrylate). Reproduced with permission.^[^ ^116^ ^]^ Copyright 2020, American Chemical Society. b) self‐heating rate from accelerated rate calorimetry (ARC) of LiFePO_4_/Li pouch cell with solid polymer electrolyte (polyether with LiTFSI) and c) with liquid carbonate‐based electrolyte at 50% and 100% state‐of‐charge. b,c) Reproduced with permission.^[^ ^118^ ^]^ Copyright 2017, Elsevier Ltd. d) schematic of a self‐crosslinking polymer electrolyte design when the temperature is increased. Reproduced with permission.^[^ ^119^ ^]^ Copyright 2019, Royal Society of Chemistry.

Although there are significant amounts of liquid electrolyte (>40 wt%) in gel‐type and quasi‐SSE systems, exothermic reactions at elevated temperature seem to be hindered to some degree. For example, differential scanning calorimetry up to 300 °C performed by Park et al.^[^ ^116^ ^]^ revealed a significant decrease in the exothermic peak observed in a NMC 622 cathode system as shown in **Figure** **6**a. In the system with liquid electrolyte a peak at 248.2 °C of 520 J g^−1^ while the quasi‐SSE with added polymer scaffold (LiTFSI, dimethyl carbonate, polycaprolactone triacrylate) yielded a small 135.9 J g^−1^ at 272.7 °C. However, in this test, the sample was merely scrapped‐off‐delithiated NMC 622 with the quasi‐SSE, that is, material level test. As safety is overwhelmingly the biggest rationale for moving from liquid to solid, it is crucial to point out that more rigorous and practically relevant thermal stabilities studies should be performed to truly clarify the precise enhancement in thermal performance between gel‐type and liquid electrolytes. Such a test could be the well‐known accelerated rate calorimetry (ARC) where pouch‐cell levels (at least 1 Ah with delithiated cathode with Li metal anode) samples are used.^[^ ^117^ ^]^ One particular study of interest^[^ ^118^ ^]^ conducted an ARC test on a pouch cell (12 cm^2^). The solid polymer electrolyte demonstrated significantly increased onset temperature (247 °C for solid vs 90 °C for liquid electrolyte) and decreased maximum self‐heating rate (below 0.2 °C min^−1^ with polymer electrolyte to >1 °C min^−1^, Figure 6b,c, respectively). However, the cathode was LFP, which is much less reactive than the more popular and energy dense Ni‐rich cathode materials and the total capacity of the cell was not reported.

Interestingly, cross‐linking of the polymer electrolyte at high temperatures can be an interesting mechanism toward a built‐in safety functionality. Crosslinking at higher temperature of the polymer electrolyte would be essentially a shut‐off of the battery (Figure 6d). With the cathode and anode physical separated with no ion transport channels, the possibility of a thermal runaway is reduced. This has been demonstrated using lithium iodide and poly(vinylene carbonate), where the cell are automatically shut down (electrolyte crosslinked) at 80 °C, resulting in a few orders of magnitude of increase in cell impedance.^[^ ^119^ ^]^ While this temperature is rather low, conceptually, a gel electrolyte could be synthesized with cross‐linking temperature tuned higher.

Furthermore, the combined use of just ionic liquid and ceramic particles, which has been shown to possess great potential in thermal performance. Ionic liquids are significantly less flammable than organic solvents and therefore, should impose a higher thermal stability. One particularly example entails the use of room temperature ionic liquid‐infused bentonite.^[^ ^120^ ^]^ From TGA analysis, this electrolyte systems only began to exhibit mass loss at 355 °C and also has stable cycling at 120 °C in a Li_4_Ti_5_O_12_ vs Li metal cell. Unfortunately, oxidative decomposition was observed at only 3.1 V vs Li^+^/Li from linear sweep voltammetry at 120 °C, making it incompatible with more common higher voltage cathode such as NMC, LCO, etc.

#### 4.4.2 All‐Solid‐State Electrolyte

Use of liquid‐state electrolyte in its entirety or as a component in near‐solid systems will inevitably vaporize under high temperature, present possibility of leakage, and undergoes severe side reaction with electrodes over time. True solid‐state systems without the assistance of liquid electrolytes have been pursued for many years.^[^ ^121^ ^]^ Recent years, liquid‐free SSEs have shown significant advances in Li‐ion conductivity to levels that are competitive with liquid organic electrolytes at room temperature and improve with increased operating temperatures.^[^ ^122^ ^]^ With a high melting point, thermal runaways and electro‐chemical reactivity are significantly reduced (due to lack of any vaporizable organic electrolyte, that is, fuel), making liquid‐free solid‐state systems very attractive for high temperature operation.^[^ ^123^ ^]^ SSEs (such as the garnet class) have high resistance against decomposition when under direct contact with Li metal.^[^ ^122^ ^]^ The use of liquid‐free SSE is likely one of the best options for high temperature batteries. Garnet‐based SSE (V_2_O_5_ cathode and Li metal anode) have demonstrated good cycle stability at 100 °C and was not ignitable in pellet form.^[^ ^123b^ ^]^ The ionic conductivity increased from 3.7 × 10^−4^ S cm^−1^ at room temperature to 2.3 × 10^−3^ S cm^−1^ at 100 °C, easily reaching conductivities in the liquid electrolyte regime.

**Figure 7:**
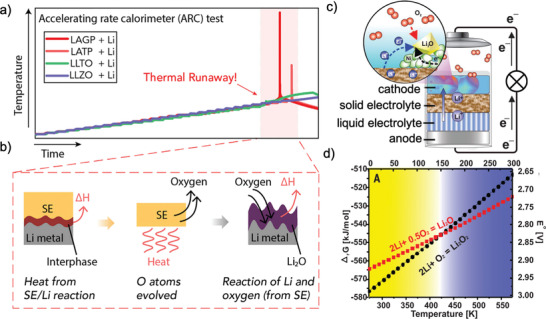
a) ARC test of various SSE, b) schematic of the thermal runaway mechanism. a,b) Reproduced with permission.^[^ ^125^ ^]^ Copyright 2020, Elsevier. c) schematic of high temperature Li‐O_2_ enabled by SSE and d) Gibbs free energy of reaction as a function of temperature, indicating the possibility of a four electron process at elevated temperature (≈150 °C). c,d) Reproduced with permission.^[^ ^130^ ^]^ Copyright 2018, AAAS.

While it might be difficult to trigger, thermal runaways are still possible for liquid‐free SSE as the reaction between SSE and Li metal are exothermic in nature.^[^ ^124^ ^]^ ARC study on various types of SSEs with Li metal have demonstrated that thermal runaways are still possible (**Figure** **7**a).^[^ ^125^ ^]^ It was explained that O_2_ gas could have been generated (which was supported by theoretical calculations) from the SSE, contributing to the thermal runaway. The decomposition products (including O_2_ gas) formed at the interface between the SSE and Li metal were expected to be accelerated at high temperature and in turn was believed to propagate the thermal runaway reaction (Figure 7b). However, no O_2_ was detected experimentally, which could be simply due to the rapid nature of the reaction between O_2_ and Li at high temperatures. Out of the tested SSEs (Li_1.5_Al_0.5_Ge_1.5_(PO_4_)_3_, Li_1.4_Al_0.4_Ti_1.6_(PO_4_)_3_, Li_3_ *_x_*La_2/3‐_ *_x_*TiO_3_, and Li_6.4_La_3_Zr_1.4_Ta_0.6_O_12_ (LLZO), Only LLZO (garnet‐type SSE) did not exhibit any significant thermal runaway (self‐heating). It was well known that the reaction of LLZO with Li metal have a low thermodynamic driving force in comparison to the other tested SSEs (Figure 7c).^[^ ^126^ ^]^ Thermal runaways might also be expected for sulfide‐type SSEs where PS_4_^3−^ are typically present, which were even less stable than phosphates.

Similar to elevated temperature cycling of Li metal in liquid organic electrolytes, liquid‐free systems SSEs could also have enhanced Li deposition kinetics.^[^ ^127^ ^]^ In liquid systems, the SEI formed at 60 °C was found to be more robust than that of the room temperature‐formed SEI.^[^ ^127^ ^]^ Li deposits were also found to be larger with increasing temperature. This is likely due to the tendency of surface energy to decrease with temperature and the subsequent inability of the LMA/electrolyte interface to maintain high curvatures (i.e., small spherical deposits), according to the solid/liquid‐modified Young‐Laplace equation.^[^ ^128^ ^]^ Such benefits might be transferable for solid SSE/solid Li metal systems. It is currently mostly unexplored whether or not the enhanced decomposition kinetics of SSE when in contact with Li metal would be more passivating or more parasitic. Furthermore, this would also likely highly depend on the nature (ionic and electronic conductivity^[^ ^129^ ^]^) of the SSE/Li metal decomposition interface. More work is required to understand the extent of the often‐claimed safety benefits of using SSEs.

In addition to the obvious benefits of increasing ionic conductivity and the likely safety benefits of using liquid‐free SSEs over liquid containing electrolytes, there are additional benefits that are unlocked at elevated operating conditions for Li metal using liquid‐free SSEs. The performances of liquid‐free SSE at room temperature and at elevated temperatures both suffer from the same interfacial contact problems that are present for room temperature SSE systems. Accordingly, contact‐enhancing layers have been often used to reduce this problem of garnet‐type SSEs with Li metal.^[^ ^131^ ^]^ One particular example of interest is the use of Li/Mg alloy for garnet‐type SSE. Mg was sputtered onto the garnet‐type SSE as a precursor of the contact layer for Li metal. A pre‐cycling heat treatment step (at 300 °C) was required to alloy the Mg with Li metal.^[^ ^132^ ^]^ Stable performance in a symmetric cell was achieved for over 35 hours of total cycling albeit at only 0.1 mA cm^−2^. More importantly, in a recent study, a sulfide‐type SSEs (Li_3_PS_4_) was also reported to benefits significantly from a similar deposited contact‐layer. Specifically, a thin layer (≈60 nm) of Au have demonstrated enhanced cycle performance and it was suggested that when the cell was operated at 100 °C, Au *in‐situ* diffused into and alloyed with Li at the Li/Li_3_PS_4_ interface, possibly limiting the undesirable formation of interfacial voids. Pairing with an NMC 111 cathode, decent performance was obtained at a relevant current density of 1.3 mA cm^−2^ and a large areal capacity of 6.5 mAh cm^−2^ at only 100 °C (in comparison to the 300 °C pretreatment step required for the Mg layer), but for only 5 cycles.^[^ ^133^ ^]^

Liquid‐free SSE can also offer itself as a reliable physical barrier, preventing chemical crossover. One excellent example of this is the high temperature operation of Li‐O_2_ battery. Nazar et al.^[^ ^130^ ^]^ demonstrated the use of SSE (Li_1.5_Al_0.5_Ge_1.5_(PO_4_)_3_) as the physical barrier separating the cathode and anode for high temperature (150 °C) Li‐O_2_ battery. It should be noted that the eutectic molten blend of LiNO_3_/KNO_3_ was used as the electrolyte contacting the electrodes to facilitate the complicated O_2_ conversion reaction to Li_2_O Through this unique configuration, lab‐scale four electron electrochemical reduction of O_2_ to Li_2_O was made thermodynamically possible by operating at elevated temperatures (Figure 7d). Additionally, O_2_ cross‐over to the Li metal anode was prevented by the SSE layer.^[^ ^130^ ^]^ These works highlight just two unique possibility that are enabled from cycling at a higher operating temperature.

## 5 Summary and Outlook

As society progresses and technology develops, battery scientists and engineers must continue to invent and develop new battery technologies capable of enabling the next generation of powered devices and applications. Innovation and deployment of LBs can serve as the buffer to mitigate energy crisis and environmental pollution problems owing to their high energy density and natural abundance. LMA is one of the most promising anode technologies moving forward, but its extreme temperature performance must be investigated and optimized in a manner which also addresses its critical issues in stability and safety. Moreover, beyond just retaining that focus with LMA, it will also be important to illuminate the key role of electrolyte and its advantages for extreme temperatures operation. Understanding how Li salts, electrolyte component and additives affect the charge/mass transfer kinetics and thermodynamic stability of liquid electrolyte is able to guide the electrolyte design toward superior performance. Meanwhile, the investigation of solid‐state electrolytes for low and high temperature operation points out a new pathway for electrolyte design, which also clearly identifies the significance to develop SSE for next generation wide‐temperature and high safety LBs. Apart from that, the LMA‐based batteries, including LMBs, Li‐S batteries and lithium‐O_2_ batteries, all illustrate broad frameworks for thinking about its versatility, such as high energy efficiency, cyclic stability, environmental benignity and cost‐effectiveness. Each of these battery chemistries present new paradigms and considerations for extreme temperature electrolyte design, but each also present unique hurdles toward widescale adoption. As for the practical rechargeable batteries, the high energy density requirement (>300 Wh kg^−1^) and rate performance under low temperature operation condition is difficult to meet based on the present technology. Meanwhile, the cyclic stability (>500 cycles) and high safety under raised temperature operation condition has not been accomplished yet. Searching for new electrolyte to improve electrolyte performance is highly desired for both research investigation and industrial application. Some strategies have great promises to be used for practical low temperature batteries such as local high concentration electrolyte, all fluorinated electrolyte, etc. Moreover, the SSE will play a key role for extreme temperature batteries application. Overall, developing advanced electrolyte for LBs in extreme temperature service scenario is pivotal for battery research, but still in its infancy toward practical application.

## Conflict of Interest

The authors declare no conflict of interest.
